# Multiple-Ring Enhancing Lesions in an Immunocompetent Adult

**DOI:** 10.4103/0974-777X.68545

**Published:** 2010

**Authors:** Amit Agrawal, Arvind Bhake, VM Sangole, Brij R Singh

**Affiliations:** *Dr. Amit Agrawal (MCh): Professor in Neurosurgery, Department of Neurosurgery, Datta Meghe Institute of Medical Sciences, Sawangi (Meghe), Wardha (India)*; *Dr. Arvind Bhake (MD): Professor in Pathology, Department of Pathology, Datta Meghe Institute of Medical Sciences, Sawangi (Meghe), Wardha (India)*; *Dr. V.M. Sangole (MD): Professor in Pathology, Department of Pathology, Datta Meghe Institute of Medical Sciences, Sawangi (Meghe), Wardha (India)*; *Dr. Brij R Singh (DMRD): Assistant Lecturer in Radiology, Department of Radiology, Datta Meghe Institute of Medical Sciences, Sawangi (Meghe), Wardha (India)*

Sir,

*Toxoplasma gondii* (an intracellular coccidian parasite) is the most common cause of protozoon infections in humans and is highly prevalent in the adult population in the world and demonstrates various clinical manifestations throughout the world.[1–3] A 25-year-old gentleman was a known case of seizure disorders for the last 8 months and was receiving anticonvulsants regularly. He was diagnosed as having a right frontal ring enhancing lesion, a positive Mantoux test (14 × 15 mm with erythema) and a raised erythrocyte sedimentation rate (18 mm in the first hour). There was no history of loss of appetite, loss of weight and lack of energy or history of alcohol addiction or blood transfusion. General physical examination, including abdominal examination and pulmonary and cardiac examinations was normal. There was no lymphadenopathy. Based on imaging findings and laboratory findings in an endemic setting of a developing country, a diagnosis of cerebral tuberculoma was suspected and the patient was started on anticonvulsants and antitubercular treatment at a peripheral hospital. However, the follow-up magnetic resonance imaging (MRI) scan showed that the lesion was persistent and there was appearance of new lesions. MR spectroscopy showed significant elevation of lipid peak with decreased N-acetyl aspartate (NAA) choline and creatinine peak. Human immunodeficiency virus (HIV) was nonreactive on enzyme-linked immunosorbent assay (ELISA). Liver function was normal. On MRI, there were multiple thick wall ring enhancing lesions in bilateral frontal lobes. The largest one was seen in the right frontal lobe, measuring 2.4 × 2.0 × 2.4 cm in the subcortical location with significant perilesional vasogenic edema. On the left side, the lesions were cortical based, which were symmetrical in size. On T2/ Fluid attenuated-inversion-recovery (FLAIR), the lesions were hypointense with central hyperintensity signal. None of the lesions showed restriction of diffusion on DWI sequence. Single multivoxel MR spectroscopy in the right frontal lobe lesion at TE 35/144 showed significant elevation of lipid peak with a decrease in NAA choline and creatinine peaks [Figure 1]. All these findings were suggestive of likely infective disease. Following these findings, a diagnosis of neurocysticercosis was also suspected and Tab Albendazole was added. The patient was investigated for the immunocompromised state and the serological tests for HIV were negative. Also, ELISA for anticysticercosis antibodies and polymerase chain reaction for *Mycobacterium tuberculosis* antigen were negative. His chest X-ray and electrocardiography were normal. A repeat contrast MRI after 9 months showed the persistence of all the lesions and the patient was referred for a biopsy of the lesions. The patient underwent right frontal craniotomy and excision of the right frontal lesion. During surgery, there was well-defined firm, thick-walled lesion containing thick yellowish material. Histopathological examination of the specimen showed thick wall abscess and necrotic tissue with inflammatory cells in the center, features suggestive of toxoplasma cerebral abscess [Figure 2]. Further history of the patient revealed that there were many cats near his residence. The patient was started on appropriate treatment and was doing well at follow-up. Human infection is usually oligosymptomatic in immunocompetent individuals and the disease is considered to be self-limited and does not require treatment.[1,3,4] Severe acute disseminated toxoplasmosis has been rarely described in immunocompetent patients and,[5,6] occasionally in normal patients, wherein they can develop long-lasting symptoms.[7,8] The most common histological finding of toxoplasma abscess is nonspecific coagulative necrosis without toxoplasmic cysts.[1,9] Although the gross pathology of a cerebral toxoplasmic abscess may resemble a pyogenic abscess, there are distinct histopathologic differences between the pyogenic and toxoplasma abscesses. When toxoplasmosis invades the brain, particularly in an immunocompromised patient, there is central necrosis with variable petechial hemorrhage that may become encircled by a ring of free tachyzoites, encysted bradyzoites and inflammatory cells.[11] As in the present case, the core of a toxoplasma abscess consists primarily of necrotic tissue and does not have the viscous, proteinaceous and inflammatory debris as seen in pyogenic abscess.[11] On imaging, the differential diagnosis of a rim-enhancing toxoplasma abscess include a primary or metastatic neoplasm, pyogenic abscess, tuberculoma, neurocysticercosis, resolving hematoma, tumefactive demyelination or radiation necrosis.[10,11] However, in many cases, the clinical history can narrow the differential diagnosis.[11] At MRI, the toxoplasma lesions are multiple, commonly located in the deep central nuclei, posterior fossa or lobar at the gray-white matter junction, with prominent associated mass effect and edema and typically show intense rim enhancement after gadolinium administration.[4,12] As in the present case, a cerebral toxoplasma abscess may have the same appearance as a pyogenic abscess on contrast-enhanced MRI. The toxoplasma lesions are hyperintense on T2- and hypointense on T1-weighted sequences.[11,12] However, unlike water diffusion in the center of a pyogenic abscess, water diffusion is not restricted in the center of the toxoplasma abscess.[11,12] Spectroscopy is characterized by a predominant lipid peak.[13] Presumptive diagnosis is considered in patients with >200 CD4+ T-lymphocyte cells/mL, anti-Toxoplasma IgG antibody in the serum, consistent clinical features, characteristic neuroimaging studies and response to empirical anti-Toxoplasma therapy. However, the definitive diagnosis can be made once there is direct demonstration of the tachyzoite form of the parasite in brain tissue.[14–16] As in the present case, rarely, the Mantoux test can be strongly positive even when the skin test for toxoplasmosis is negative.[17] The treatment of a case of cerebral toxoplasmosis includes pyrimethamin 50–100 mg/day and sulfadiazine 4 g/day. If the patient is allergic to sulfa drugs, he can alternatively be put on clindamycin 600 mg qid.[18]

**Figure 1:**
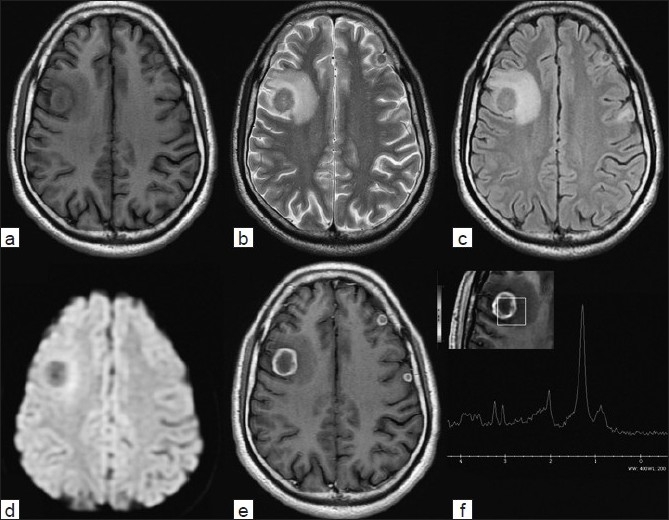
Magnetic resonance imaging of the brain showing a well-defined hypointense lesion on T1W image (a), becoming hyperintense on T2W (b), FLAIR (c) diffusion-weighted images (d). Also note the ring enhancement after gadolinium administration in (e), the spectroscopy showed the lipid peak (f)

**Figure 2:**
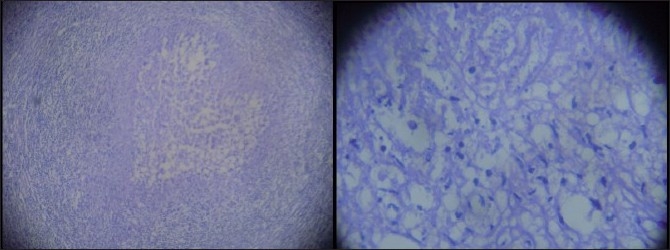
Photomicrography histopathologic specimens of cerebral toxoplasma abscess and the wall of abscess consisting of viable acute inflammatory cells, including macrophages and toxoplasma tachyzoites, and encysted bradyzoites (H and E stain left ×40, right ×100)

## References

[1] Montoya JG, Liesenfeld O (2004). Toxoplasmosis. Lancet.

[2] John DT, Petri WA (2006). Markell and Voge’s Medical Parasitology.

[3] Nissapatorn V, Lee C, Quek KF, Leong CL, Mahmud R, Abdullah KA, Mahmud R, Abdullah KA (2004). Toxoplasmosis in HIV/AIDS patients: a current situation. Jpn J Infect Dis.

[4] Habek M, Ozretić D, Zarković K, Djaković V, Mubrin Z (2009). Unusual cause of dementia in an immunocompetent host: toxoplasmic encephalitis. Neurol Sci.

[5] Bossi P, Paris L, Caumes E, Katlama C, Danis M, Bricaire F (2002). Severe acute disseminated toxoplasmosis acquired by an immunocompetent patient in French Guiana. Scand J Infect Dis.

[6] Leal FE, Cavazzana CL, de Andrade HF, Galisteo AJ, de Mendonça JS, Kallas EG (2007). Toxoplasma gondii pneumonia in immunocompetent subjects: case report and review. Clin Infect Dis.

[7] Durlach RA, Kaufer F, Carral L, Hirt J (2003). Toxoplasmic lymphadenitis -clinical and serologic profile. Clin Microbiol Infect.

[8] Moscatelli G, Altcheh J, Biancardi M, Lapeña A, Ballering G, Freilij H (2006). Acute toxoplasmosis: clinical and laboratory data in eleven patients. An Pediatr (Barc).

[9] Luft BJ, Remington JS (1992). Toxoplasmic encephalitis in AIDS. Clin Infect Dis.

[10] Nakazaki S, Saeki N, Itoh S, Osato K, Watanabe O, Hamada N (2000). Toxoplasmic encephalitis in patients with acquired immunodeficiency syndrome–fourcase reports. Neurol Med Chir (Tokyo).

[11] Chong-Han CH, Cortez SC, Tung GA (2003). Diffusion-weighted MRI of cerebral toxoplasma abscess. AJR Am J Roentgenol.

[12] Bakshi R (2004). Neuroimaging of HIV and AIDS related illnesses: a review. Front Biosci.

[13] Skiest DJ, Erdman W, Chang WE, Oz OK, Ware A, Fleckenstein J (2000). SPECT thallium-201 combined with Toxoplasma serology for the presumptive diagnosis of focal central nervous system mass lesions in patients with AIDS. J Infect.

[14] Mamidi A, DeSimone JA, Pomerantz RJ (2002). Central nervous system infections in individuals with HIV-1 infection. J Neurovirol.

[15] Skiest DJ (2002). Focal neurological disease in patients with acquired immunodeficiency syndrome. Clin Infect Dis.

[16] Cohen BA (1999). Neurologic manifestations of toxoplasmosis in AIDS. Semin Neurol.

[17] Malik SR, Gupta DK, Prakash O (1969). Ocular toxoplasmosis. J All India Ophthalmol Soc.

[18] Kastrup O, Wanke I, Maschke M (2008). Neuroimaging of infections of the central nervous system. Semin Neurol.

